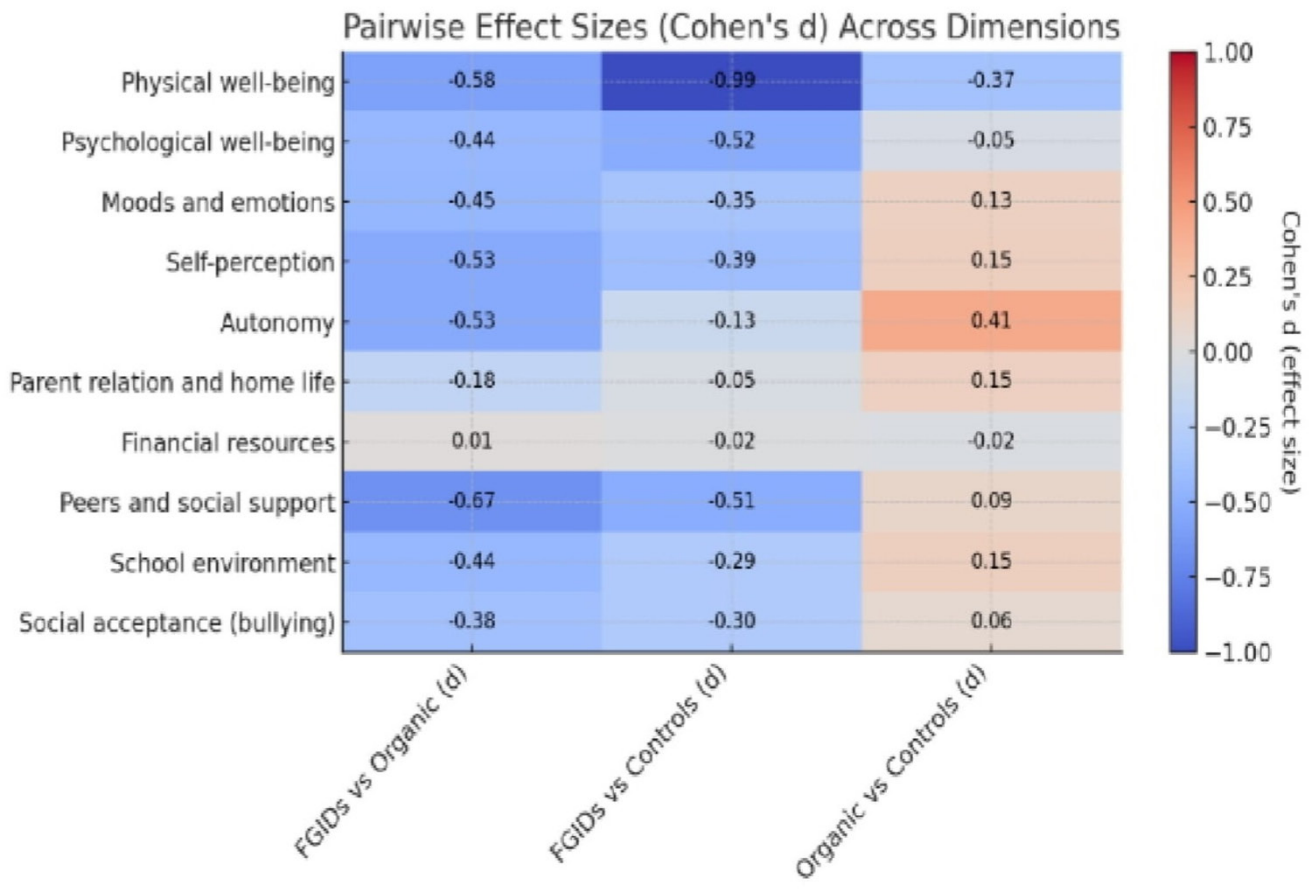# Poster Session I - A154 HEALTH-RELATED QUALITY OF LIFE IN JORDANIAN CHILDREN WITH FUNCTIONAL AND ORGANIC GASTROINTESTINAL DISORDERS: A CROSS-SECTIONAL STUDY USING THE ARABIC KIDSCREEN-52

**DOI:** 10.1093/jcag/gwaf042.154

**Published:** 2026-02-13

**Authors:** E Altamimi, H M Algharaibeh, K A Barham, R K Jaddallah, M Bataineh

**Affiliations:** Pediatrics and Neonatology, Jordan University of Science and Technology Faculty of Medicine, Irbid, Irbid Governorate, Jordan; Pediatrics and Neonatology, Jordan University of Science and Technology Faculty of Medicine, Irbid, Irbid Governorate, Jordan; Pediatrics and Neonatology, Jordan University of Science and Technology Faculty of Medicine, Irbid, Irbid Governorate, Jordan; Pediatrics and Neonatology, Jordan University of Science and Technology Faculty of Medicine, Irbid, Irbid Governorate, Jordan; Pediatrics and Neonatology, Jordan University of Science and Technology Faculty of Medicine, Irbid, Irbid Governorate, Jordan

## Abstract

**Background:**

Children with gastrointestinal (GI) disorders, particularly functional gastrointestinal disorders (FGIDs), may experience impaired health-related quality of life (HRQoL). Data from Jordan and the Arab region are limited. This study assessed HRQoL among Jordanian children with FGIDs and organic GI disorders using the Arabic KIDSCREEN-52.

**Aims:**

This study aims to assess HRQoL among Jordanian children with FGIDs and organic GI disorders using the Arabic KIDSCREEN-52 and to compare their scores to children with no gastrointestinal disorders.

**Methods:**

In this cross-sectional study, 77 children aged 8–18 years with confirmed GI disorders (35 FGIDs, 42 organic) completed the Arabic KIDSCREEN-52. HRQoL scores were compared between FGID and organic groups and with 230 healthy controls. Subgroup analyses examined gender differences. Statistical analyses included t-tests, ANOVA, and post-hoc comparisons.

**Results:**

Children with FGIDs consistently reported lower HRQoL across multiple domains compared with those with organic GI diseases. Significant differences were observed in physical well-being (45.1 ± 10.7 vs. 51.8 ± 12.1, p = 0.01), psychological well-being (48.8 ± 9.2 vs. 53.3 ± 11.1, p = 0.05), moods and emotions (44.7 ± 9.8 vs. 50.0 ± 13.3, p = 0.05), self-perception (49.1 ± 9.8 vs. 55.0 ± 12.1, p = 0.02), autonomy (48.4 ± 12.1 vs. 54.4 ± 10.6, p = 0.02), and peer/social support (47.3 ± 12.0 vs. 54.6 ± 10.1, p = 0.005). Compared with healthy peers, children with GI disorders had significantly lower physical well-being (48.8 ± 11.9 vs. 56.1 ± 11.2, p < 0.001), while other domains were similar. No significant gender differences were observed.

**Conclusions:**

FGIDs are associated with substantial impairments in HRQoL, particularly in physical and social domains, compared with organic GI disorders and healthy children. These findings highlight the need for early recognition and holistic management of FGIDs in Jordanian pediatric populations.

A154 Table 1: Comparison of Quality-of-life scores between patients with Functional Gastrointestinal Disorders and patients with Organic Gastrointestinal Disorders:

A heatmap of Cohen’s d effect sizes between the three groups across all quality-of-life dimensions shows clear patterns, where blue indicates that FGIDs score much lower than the comparison group and red indicates higher scores, with the intensity reflecting the magnitude of the effect. At a glance, physical well-being and peers/social support stand out with the largest differences, as FGIDs score markedly lower than both groups in these domains.

**Funding Agencies:**

None